# Association between a body shape index and abdominal aortic calcification in general population: A cross-sectional study

**DOI:** 10.3389/fcvm.2022.1091390

**Published:** 2023-01-10

**Authors:** Wei Li, Zhenwei Wang, Min Li, Jing Xie, Jing Gong, Naifeng Liu

**Affiliations:** ^1^Department of Cardiology, Zhongda Hospital, School of Medicine, Southeast University, Nanjing, China; ^2^Department of Cardiology, Affiliated Hospital of Yangzhou University, Yangzhou, China; ^3^College of Basic Medicine and Clinical Pharmacy, China Pharmaceutical University, Nanjing, Jiangsu, China

**Keywords:** a body shape index, body mass index, waist circumference, waist-to-height ratio, abdominal aortic calcification

## Abstract

**Background:**

The association between a body shape index (ABSI) and abdominal aortic calcification (AAC) is still unclear, so we tried to prove the association between ABSI and AAC in the general population in this cross-sectional study.

**Materials and methods:**

After excluding participants with missing data on height, weight, waist circumference (WC), and AAC, we finally selected 3,140 participants aged 40–80 years from the 2013–2014 National Health and Nutrition Examination Survey. Using multivariate logistic regression and receiver operating characteristic (ROC) curves to test the association between ABSI and AAC.

**Results:**

Participants (median age: 58.0 years; 48.3% men) were divided into two groups by the optimal cutoff point of ABSI: higher ABSI (> 0.84) and lower ABSI (≤ 0.84). Participants with higher ABSI showed significantly higher proportion of AAC than those with lower ABSI (39.8 vs. 23.7%, *P* < 0.001). Participants with higher ABSI had an increased risk of developing AAC in crude model (ABSI as a continuous variable: OR = 2.485, 95% CI: 2.099–2.942, *P* < 0.001; as a categorical variable: OR = 2.132, 95% CI: 1.826–2.489, *P* < 0.001), and ABSI was still independently associated with AAC in all adjusted models (all *P* < 0.05). Further subgroup analyses showed that higher ABSI was consistently associated with AAC in subgroups with sex (male or female), age (≤ 65 or > 65 years), smoking history (yes or no), hypertension (yes or no), diabetes (yes or no), sleep disorder (yes or no), body mass index (BMI) (< 23 or ≥ 23 kg/m^2^), systolic blood pressure (< 140 or ≥ 140 mmHg), diastolic blood pressure (< 90 or ≥ 90 mmHg), fasting plasma glucose (< 126 or ≥ 126 mg/dL), and low-density lipoprotein cholesterol (≤ 130 or > 130 mg/dL) (*P* for interaction > 0.05). While in other subgroups, the association was no longer synchronized. The ROC showed that the area under the curve of ABSI was significantly higher than height, weight, BMI, WC, and waist-to-height ratio (WHtR).

**Conclusion:**

Higher ABSI was closely associated with higher risk of AAC, and discriminant ability of ABSI for AAC was significantly higher than height, weight, BMI, WC, and WHtR.

## Background

Abdominal aortic calcification (AAC) refers to vascular calcification in the abdominal aorta, which has been proved by previous studies to be related to coronary artery calcification and the severity of cardiovascular diseases (CVDs) (1–3). Calcium deposits may occur in all layers of blood vessels, including intima, media, and adventitia (4, 5). The mechanism of vascular calcification has not been fully elucidated. It has been reported that chronic inflammation, insulin resistance, oxidative stress, vascular smooth muscle cell transdifferentiation, mitochondrial dysfunction, apoptosis, autophagy and DNA damage are involved in the occurrence of vascular calcification (4, 6–8). And in clinical practice, it has been reported that advanced age, smoking, obesity, diabetes, dyslipidemia and low relative lean mass may be the risk factors of AAC (4, 9–12). However, there may be other risk factors for AAC, such as nutritional and metabolic disorders. Further elucidation of other risk factors of AAC and targeted intervention are beneficial to prevent the occurrence and development of AAC, thereby reducing the occurrence of cardiovascular events.

For decades, with the improvement of living conditions, obesity, especially central obesity, has become an increasingly serious global health problem (13). In contrast to subcutaneous fat, visceral fat accumulation has been shown to be closely associated with dyslipidemia, insulin resistance, diabetes and hypertension, all of which increase the risk of CVDs (13–15). Hence, it is of great significance to find a propagable and simple clinical tool for detecting visceral fat and diagnosing central obesity. At present, the traditional anthropometric indicators mainly include height, weight, body mass index (BMI), waist circumference (WC), hip circumference andwaist-to-height ratio (WHtR). However, these indicators have some limitations. For instance, BMI can’t reflect the fat distribution (14). Although WC is generally considered to be a sign of central obesity, it still has some limitations, such as being disturbed by race and gender (16, 17). Therefore, in order to overcome these limitations, Krakauer et al. developed a new nutritional index, namely a body shape index (ABSI), which is calculated from height, weight and WC (18). And their study confirmed that ABSI was positively associated with visceral fat or central obesity, and they also found that the association between ABSI and premature death was higher than that of BMI and WC (18). Since then, ABSI has received more and more attention. Subsequent studies revealed the association between ABSI and arterial stiffness (19), carotid atherosclerosis (20), hypertension (21), metabolic syndrome (22), diabetes, and CVDs (23–27). For example, Ma et al. showed in a large cross-sectional study that ABSI was closely related to subclinical carotid atherosclerosis in participants without cardio-cerebrovascular disease and hypertension, diabetes, and hyperlipidemia (28). In addition, Otaki et al. found an independent association between ABSI and deaths associated with aortic disease in a large cohort study of 630,842 participants (29).

However, as far as we know, data about the association between ABSI and AAC is currently lacking. Furthermore, there are no related studies to compare the predictive efficiency of traditional anthropometric indicators and ABSI for AAC. Therefore, the present study was to explore the association between ABSI and AAC. Besides, we also tried to test the predictive ability of ABSI and anthropometric indicators for AAC in the general population aged 40–80 years from the 2013–2014 National Health and Nutrition Examination Survey (NHANES 2013–2014).

## Materials and methods

### Study population

**FIGURE 1:**
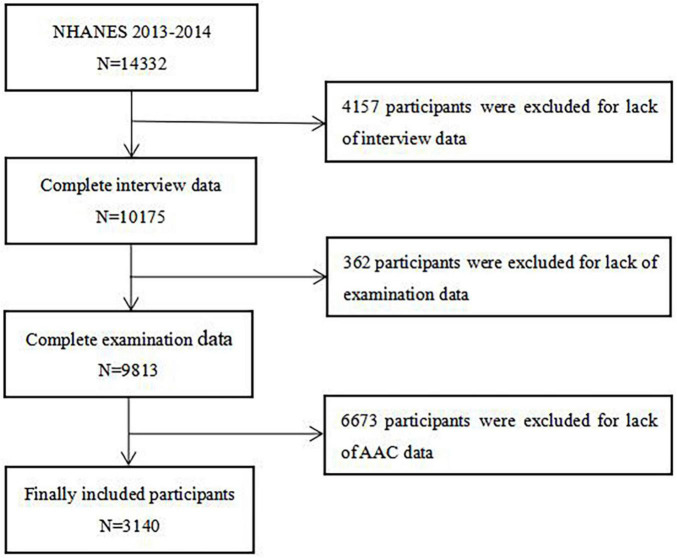
Flow chart of the study population enrollment. NHANES 2013–2014, 2013–2014 National Health and Nutrition Examination Survey; AAC, abdominal aortic calcification.

National Health and Nutrition Examination Survey (NHANES) is a regular survey of representative samples of the general population in the United States, which aimed to investigate the health and disease status of the general population in the United States and provide perfect health guidance, the contents and survey data of which have been described in detail in other literatures (30). After excluding participants with missing data on height, weight, WC, and AAC, we finally selected 3,140 participants aged 40–80 years from the NHANES 2013–2014 for this cross-sectional study. The protocol of NHANES 2013–2014 was approved by the National Center for Health Statistics of the Center for Disease Control and Prevention Institutional Review Board (Protocol #2011-17), all participants of the present study provided written informed consent at the time of enrollment, and the study was consistent with the principles of the Declaration of Helsinki. Flow chart of participant selection of the present study was shown in the figure below (Figure 1).

### Survey and measurement

The demographic characteristics of all participants were obtained by standardized family interview questionnaire, including age, sex, race, smoking history, history of diabetes, hypertension, osteoporosis, and sleep disorder. The race was divided into five groups: non-Hispanic White, non-Hispanic Black, Mexican American, other Hispanic and others. Smoking history was divided into two groups: yes and no. Height, weight, systolic blood pressure (SBP), diastolic blood pressure (DBP), and WC were measured by medical trained professionals according to measurement procedures and standards, and the calculation method of the BMI was: weight (kg) divided into the square of the height (meter). The WHtR was defined as the ratio of WC to height. The parameter values of blood samples of participants were determined strictly according to operational procedures by medically trained technicians in standard basic laboratory, including blood lipid profile, fasting plasma glucose (FPG), hemoglobin A1c (HbA1c), serum electrolytes, kidney function, etc.

For the calculation of ABSI, we used the formula described by Krakauer et al. based on height, BMI and WC (18), that is:


$$\text{ABSI}=\frac{\text{WC}}{{\text{height}}^{\frac{1}{2}}x{\mathrm{BMI}}^{\frac{2}{3}}}$$

In our study, we divided participants into two groups based on the optimal cutoff point of ABSI: higher ABSI (> 0.84; *n* = 1,264) and lower ABSI (≤ 0.84; *n* = 1,876).

**FIGURE 2:**
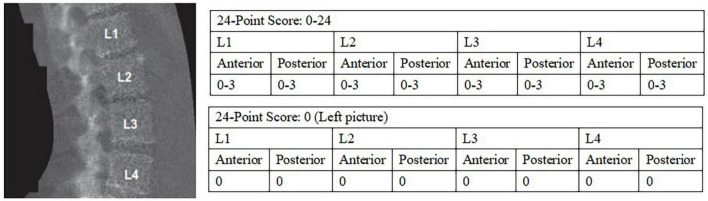
24-point semi-quantitative AAC scale and image by DXA (33). AAC, abdominal aortic calcification; DXA, dual-energy X-ray absorptiometry.

Abdominal aortic calcification (AAC) was gained by transverse scanning of the lumbar spine (vertebrae L1–L4) with dual-energy X-ray absorptiometry (DXA) (Densitometer Discovery A, Hologic, Marlborough, MA, USA) and semi-quantified by the Kauppila score system, with scores ranging from 0 to 24, and the specific scoring rules of AAC have been described in detail elsewhere, that is, as shown in Figure 2, the severity of calcification of the anterior and posterior walls of the abdominal aorta in each segment from L1-L4 was evaluated separately, and a score of 1 (< 1/3), 2 (1/3∼2/3) or 3 (> 2/3) was given according to the extent of calcification involvement in that segment, and the total score for each segment involved was the AAC score (0–24) for that patient (31–33). We divided AAC into two groups: no calcification (AAC = 0) and calcification (AAC > 0).

### Statistical analysis

All Statistical tests were performed with SPSS 19.0 (SPSS Inc., Chicago, IL, USA), MedCalc version 19.1 (MedCalc Software, Belgium) and R Programming Language (version 3.6.3, R Foundation for Statistical Computing, Vienna, Austria). Continuous variables were expressed as mean ± standard deviation or median (quartiles: Q1, Q3) depending on whether the data was normal distribution, and the independent-sample *t*-test or Mann-Whitney *U* test was used to examined the differences between the two groups. Categorical variables were presented as numbers (percentages), and chi-square test or fisher’s exact test was used to tested the differences between groups. The effect of ABSI on AAC was evaluated by the multivariate logistic regression in different models, including crude model and adjusted models. Crude model: unadjusted; Model 1: adjusted for age, smoking history, hypertension, diabetes, and osteoporosis; Model 2: adjusted for variables included in Model 1 and BMI, SBP; Model 3: adjusted for variables included in Model 2 and triglycerides (TG), total cholesterol (TC), creatinine, FPG. In multivariate logistic regression analysis, four models (crude model and Model 1–3) including covariables with *P* < 0.1 for avoiding missing some important factors and clinical significance were established to assess the predictive significance of ABSI for AAC. Further subgroup analyses were performed to test the consistence of the predictive significance of ABSI for AAC according to sex (male or female), age (≤ 65 or > 65 years), smoking history (yes or no), hypertension (yes or no), diabetes (yes or no), osteoporosis (yes or no), sleep disorder (yes or no), BMI (< 23 or ≥ 23 kg/m^2^), SBP (< 140 or ≥ 140 mmHg), DBP (< 90 or ≥ 90 mmHg), FPG (< 126 or ≥ 126 mg/dL), HbA1c (< 6.5 or ≥ 6.5%), and low-density lipoprotein cholesterol (LDL-C) (≤ 130 or > 130 mg/dL). The model used in the subgroup analyses did not contain other covariates. Besides, possible modifications of the association between ABSI and AAC were also assessed by interaction tests. C-statistics derived from receiver-operating characteristic (ROC) curve analysis was used to test the predictive potential of ABSI and traditional anthropometric indicators for AAC, and examine the incremental effects of ABSI on the predictive potential of the baseline risk model that including age, smoking history, diabetes, hypertension, osteoporosis, SBP, TG, TC, FPG, HbA1c, creatinine, uric acid, alkaline phosphatase (ALP), total calcium, and 25-OH-VitD3. DeLong’s test was performed to compare the area under the curve (AUC) of each prediction model. The optimal cutoff point of ABSI for predicting AAC was determine by ROC curve analysis. A two-tailed *P* value < 0.05 was regarded as statistically significant.

## Results

**FIGURE 3:**
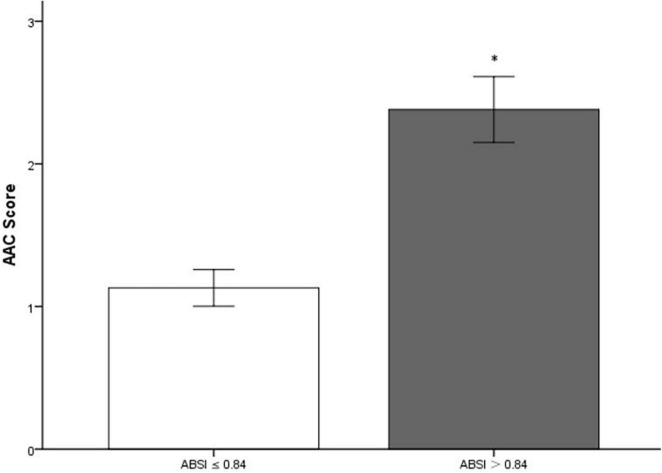
Bar graph of mean value of AAC score stratified by a body shape index (ABSI). AAC, abdominal aortic calcification. *Compared with lower a body shape index (ABSI) group, participants in higher ABSI group had significantly higher AAC score (*P* < 0.001).

The 3,140 participants [age: 58.0 (48.0, 68.0) years; 48.3% men] enrolled in the present study were divided into two groups based on the optimal cutoff point of ABSI: higher ABSI (> 0.84; *n* = 1,264) and lower ABSI (≤ 0.84; *n* = 1,876). Baseline characteristics of total population and participants stratified by the ABSI of 0.84 were displayed in Table 1 and Figure 3. Compared with participants in lower ABSI group, those with higher ABSI appeared to be older, displayed higher levels of WC, WHtR, SBP, and AAC score, and higher percentage of male, non-Hispanic White and smoker, and higher prevalence of diabetes, hypertension, osteoporosis, sleep disorder, and AAC, while lower BMI. Laboratory indices including FPG, TG, blood urea nitrogen (BUN), uric acid, creatinine, HbA1c, γ-glutamyl transpeptidase (GGT), and ALP were significantly higher in participants with higher ABSI, while TC, LDL-C, and high-density lipoprotein cholesterol (HDL-C) levels were comparatively lower (*P* < 0.05).

**TABLE 1:** Participants characteristics stratified by the optimal cutoff point of a body shape index (ABSI).

Variables	Total population (*n* = 3,140)	Lower ABSI (≤ 0.84; *n* = 1,876)	Higher ABSI (> 0.84; *n* = 1,264)	*P* value
Age, years	58.0 (48.0, 68.0)	53.0 (46.0, 63.0)	64.0 (56.0, 73.0)	< 0.001
Sex, male, n (%)	1518 (48.3)	821 (43.8)	697 (55.1)	< 0.001
Race, n (%)				< 0.001
Non-Hispanic white	1375 (43.8)	724 (38.6)	651 (51.5)	
Non-Hispanic black	620 (19.7)	439 (23.4)	181 (14.3)	
Mexican-American	412 (13.1)	253 (13.5)	159 (12.6)	
Other Hispanic	298 (9.5)	187 (10.0)	111 (8.8)	
Others	435 (13.9)	273 (14.6)	162 (12.8)	
Smoking history, n (%)	1452 (46.2)	765 (40.8)	687 (54.4)	< 0.001
Diabetes, n (%)	648 (20.6)	290 (15.5)	358 (28.3)	< 0.001
Hypertension, n (%)	1486 (47.3)	782 (41.7)	704 (55.7)	< 0.001
Osteoporosis, n (%)	258 (8.2)	127 (6.8)	131 (10.4)	< 0.001
Sleep disorder, n (%)	336 (10.7)	166 (8.8)	170 (13.4)	< 0.001
Body mass index, kg/m^2^	28.4 ± 5.6	28.8 ± 5.8	28.0 ± 5.1	< 0.001
Waist circumference, cm	99.3 ± 13.6	96.2 ± 13.0	103.9 ± 13.0	< 0.001
WHtR	0.6 ± 0.1	0.6 ± 0.1	0.6 ± 0.1	< 0.001
SBP, mmHg	127.2 ± 18.3	125.1 ± 17.1	130.4 ± 19.4	< 0.001
DBP, mmHg	71.3 ± 10.8	72.0 ± 10.2	70.3 ± 11.5	0.221
**Laboratory results**				
Triglycerides, mg/dL	132.0 (86.0, 192.8)	121.0 (80.0, 176.0)	144.0 (97.0, 211.8)	< 0.001
Total cholesterol, mg/dL	196.0 ± 42.7	198.0 ± 41.9	193.2 ± 43.7	0.002
LDL-C, mg/dL	114.8 ± 36.0	117.0 ± 35.2	111.4 ± 37.1	0.004
HDL-C, mg/dL	54.1 ± 16.5	55.6 ± 16.9	51.8 ± 15.6	< 0.001
Blood urea nitrogen, mg/dL	14.3 ± 6.2	13.6 ± 5.3	15.3 ± 7.1	< 0.001
Creatinine, mg/dL	0.9 (0.7, 1.0)	0.9 (0.7, 1.0)	0.9 (0.8, 1.1)	< 0.001
Uric acid, mg/dL	5.5 ± 1.4	5.3 ± 1.3	5.6 ± 1.4	< 0.001
FPG, mg/dL	98.0 (90.0, 110.0)	96.0 (89.0, 109.0)	102.0 (91.0, 119.0)	< 0.001
Hemoglobin A1c, %	5.7 (5.4, 6.0)	5.6 (5.3, 5.9)	5.8 (5.4, 6.2)	< 0.001
Total bilirubin, mg/dL	0.6 ± 0.3	0.6 ± 0.3	0.6 ± 0.3	0.663
GGT, U/L	21.0 (15.0, 30.0)	20.0 (14.0, 30.0)	21.0 (15.0, 31.0)	0.002
Alkaline phosphatase, IU/L	65.0 (53.0, 77.0)	64.0 (53.0, 75.0)	67.0 (54.0, 80.0)	< 0.001
Total calcium, mg/dL	9.5 ± 0.4	9.4 ± 0.4	9.5 ± 0.3	0.226
Phosphorus, mg/dL	3.8 ± 0.6	3.8 ± 0.6	3.8 ± 0.6	0.164
25-OH-VitD3, nmol/L	63.8 (45.9, 81.0)	63.3 (46.2, 79.6)	64.9 (45.7, 83.1)	0.065
AAC, n (%)	947 (30.2)	444 (23.7)	503 (39.8)	< 0.001

ABSI, a body shape index; WHtR, waist-to-height ratio; SBP, systolic blood pressure; DBP, diastolic blood pressure; LDL-C, low-density lipoprotein cholesterol; HDL-C, high-density lipoprotein cholesterol; FPG, fasting plasma glucose; GGT, γ-glutamyl transpeptidase; AAC, abdominal aortic calcification.

**FIGURE 4:**
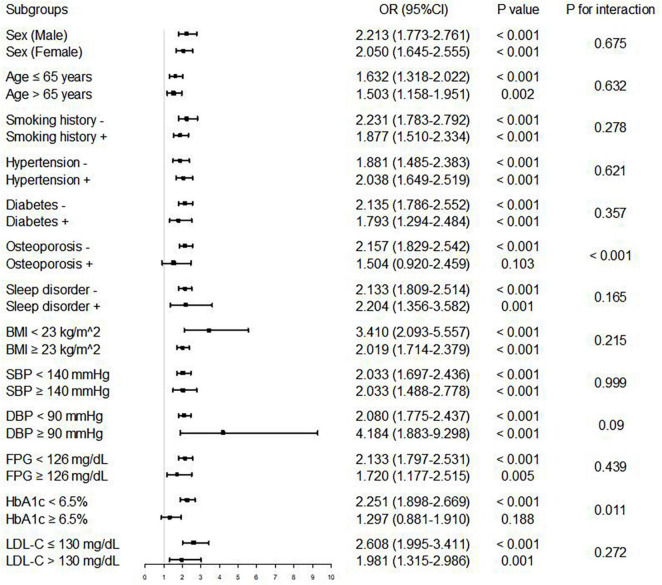
Logistic regression analysis of associations between ABSI and AAC in different subgroups. The OR was examined regarding lower ABSI as reference (stratified by the optimal cutoff point of ABSI determined by ROC curve analysis). *P* value < 0.05 and *P* for interaction < 0.05 were regarded as statistically significant. ABSI, a body shape index; BMI, body mass index; SBP, systolic blood pressure; DBP, diastolic blood pressure; FPG, fasting plasma glucose; HbA1c, hemoglobin A1c; LDL-C, low-density lipoprotein cholesterol; OR, odds ratio; CI, confidence interval.

In multivariate logistic regression analysis, with the increase of confounding factors, higher ABSI remained to be an independent risk predictor of AAC, whether ABSI was regarded as a categorical or continuous variable (all *P* < 0.05 in Model 1–3) (Table 2). Further subgroup analyses (Figure 4) showed higher ABSI (regarding lower ABSI as reference) was consistently correlated with AAC in eleven subgroups, including sex, age, smoking history, hypertension, diabetes, sleep disorder, BMI, SBP, DBP, FPG, and LDL-C (*P* for interaction > 0.05). However, in the osteoporosis and HbA1c subgroups, the association was no longer synchronized (*P* for interaction < 0.05). Interestingly, the risk of participants with higher ABSI developing into AAC seemed to be more noticeable in participants without osteoporosis [OR (95% CI): 2.157 (1.829–2.542), *P* < 0.001] and with HbA1c < 6.5% [OR (95% CI): 2.251 (1.898–2.669), *P* < 0.001].

**TABLE 2:** Univariable and multivariable logistic regression analyses of associations between a body shape index (ABSI) and abdominal aortic calcification (AAC).

	ABSI as a continuous variable^a^			ABSI as a categorical variable^b^		
	**OR**	**95% CI**	***P* value**	**OR**	**95% CI**	***P* value**
Crude model	2.485	2.099–2.942	< 0.001	2.132	1.826–2.489	< 0.001
Model 1	1.378	1.150–1.653	0.001	1.257	1.057–1.494	0.010
Model 2	1.287	1.070–1.547	0.007	1.220	1.025–1.453	0.025
Model 3	1.259	1.046–1.516	0.015	1.201	1.008–1.430	0.041

Crude model: unadjusted.

Model 1: adjusted for age, smoking history, hypertension, diabetes, and osteoporosis.

Model 2: adjusted for variables included in Model 1 and body mass index, systolic blood pressure.

Model 3: adjusted for variables included in Model 2 and triglycerides, total cholesterol, creatinine, and fasting plasma glucose.

ABSI, a body shape index; AAC, abdominal aortic calcification; OR, odds ratio; CI, confidence interval.

^a^The OR was examined by per 1-unit increase of ABSI.

^b^The OR was examined regarding lower ABSI as reference (stratified by the optimal cutoff point of ABSI determined by ROC curve analysis).

**FIGURE 5:**
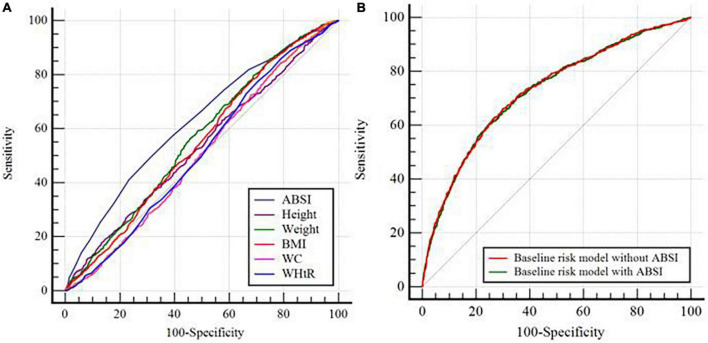
C-statistics evaluating incremental effect of different models. **(A)** ABSI vs. Height or Weight or BMI or WC or WHtR; **(B)** Baseline risk model without ABSI vs. Baseline risk model with ABSI. ABSI, a body shape index; BMI, body mass index; WC, waist circumference; WHtR, waist-to-height ratio. The baseline risk model included age, smoking history, diabetes, hypertension, osteoporosis, systolic blood pressure, triglycerides, total cholesterol, fasting plasma glucose, hemoglobin A1c, creatinine, uric acid, alkaline phosphatase, total calcium, and 25-OH-VitD3.

The ROC curve analysis showed that the discriminant ability of ABSI for AAC was significantly higher than that of other univariate predictive models, including height, weight, BMI, WC, and WHtR (all *P* for comparison < 0.001). However, the addition of ABSI had no significant increasing effect on the AUC obtained by the baseline risk model composed of age, smoking history, diabetes, hypertension, osteoporosis, SBP, TG, TC, FPG, HbA1c, creatinine, uric acid, ALP, total calcium, and 25-OH-VitD3 (AUC: baseline risk model, 0.726 vs. baseline risk model + ABSI, 0.728, *P* for comparison = 0.098) (Table 3, Figure 5).

**TABLE 3:** C-statistics for discrimination ability of different models.

Variables	AUC	95% CI	*P* value	*Z* value	*P* for comparison
**Univariate model**					
ABSI	0.625	0.608–0.642	< 0.001	Reference	Reference
Height	0.532	0.514–0.549	0.005	5.710	< 0.001
Weight	0.562	0.545–0.580	< 0.001	4.217	< 0.001
BMI	0.548	0.530–0.565	< 0.001	5.385	< 0.001
WC	0.505	0.487–0.523	0.652	6.841	< 0.001
WHtR	0.512	0.494–0.529	0.284	8.687	< 0.001
**Combined variable model**					
Baseline risk model^a^ without ABSI	0.726	0.710–0.741	< 0.001	Reference	Reference
Baseline risk model^a^ with ABSI	0.728	0.712–0.744	< 0.001	1.657	0.098

ABSI, a body shape index; BMI, body mass index; WC, waist circumference; WHtR, waist-to-height ratio; AUC, area under the curve; CI, confidence interval. ^a^The baseline risk model included age, smoking history, diabetes, hypertension, osteoporosis, systolic blood pressure, triglycerides, total cholesterol, fasting plasma glucose, hemoglobin A1c, creatinine, uric acid, alkaline phosphatase, total calcium, and 25-OH-VitD3.

## Discussion

As far as we know, the present study was the first report on the association between ABSI and AAC. In the present study, we retrospectively explored the predictive importance of ABSI for AAC. The main findings were as follows: (1) compared with participants in lower ABSI group, those with higher ABSI showed higher AAC score and higher prevalence of AAC; (2) higher ABSI increased the risk of AAC by 20–30% compared with lower ABSI, although after adjusting for possible interference factors; (3) the risk of participants with higher ABSI developing into AAC seemed to be more noticeable in participants without osteoporosis and with HbA1c < 6.5%; (4) the discriminant ability of ABSI for predicting AAC was significantly higher than height, weight, BMI, WC, and WHtR. These results suggested that ABSI may be essential for risk management of AAC.

Abdominal aortic calcification (AAC) has been widely considered as an important risk factor for CVDs, and it is very common in patients with CVDs. Some studies have shown that AAC is significantly associated with incident myocardial infarction (2), stroke (34), osteoporosis (35), fracture (36), CVDs mortality (2), and all-cause mortality (3). Therefore, the identification of pathogenic factors of AAC is of great clinical significance for primary and secondary prevention of CVDs. Several studies showed that advanced age, smoking, diabetes, obesity, and dyslipidemia may be the risk factors of AAC (4, 9–11). However, there may be other risk factors for AAC, such as nutrition indices.

At present, BMI and WC are the most commonly used anthropometric indicators in clinical practice, but both of them have some limitations in fat distribution. First, BMI not only can’t distinguish between adipose and non-adipose tissue, but also can’t reflect the distribution of fat (37). In fact, people with excess visceral fat or central obesity are more likely to develop CVDs and metabolic syndrome (14, 15). Unlike BMI, WC has always been regarded as an alternative indicator of central obesity (38). Previous studies have shown that WC could predict the risk of death better than BMI (39, 40), but a comparative study showed that WC was weakly or negatively correlated with subclinical CVDs (19). This suggests that the ability of WC to predict metabolic-related diseases may be overrated. The reason for this may be that the WC can’t distinguish between subcutaneous fat and visceral fat, and can’t reflect the difference of height and race (17). Therefore, WC may not be enough to fully represent central obesity. In addition, although there is evidence that WHtR derived from WC and height can predict metabolic disorders (41), it fails to reflect differences of weight between individuals. Therefore, it is essential to develop a better tool to assess central obesity. It is reported that imaging technology is the gold standard for the evaluation of central obesity, but it is difficult to be widely popularized because of its high cost, complex operation, and radiation. Therefore, a simple evaluation method comes into being, that is, ABSI developed by Krakauer et al. in 2012 (18).

The association between a body shape index (ABSI) is a recently developed nutritional index composed of height, weight, and WC, which is reported to be positively associated with central obesity, metabolic related diseases and death risk (18, 22). A subsequent study found that among teenagers, ABSI was better in identifying hypertension than BMI and WC (42). And in Chinese adults, ABSI is a better predictor of diabetes and metabolic syndrome than BMI and WC (25). Recent studies have also found that ABSI had a stronger association with all-cause mortality and CVDs mortality than WC, BMI, and WHtR, and it might be an important marker of atherosclerosis in patients with type 2 diabetes (43, 44). Similarly, a study by Geraci et al. has also shown that ABSI might be a better predictor of carotid atherosclerosis in patients with hypertension than traditional nutrition indexes, including WC and BMI (20). However, some studies have found that ABSI is not superior to BMI and WC in predicting the risk of related disease or death. For example, two studies coincidentally found that in Chinese children, adolescents or adults and the elderly, the association between ABSI and pre-hypertension or hypertension was not higher than WC, BMI and WHtR, and the WHtR had the highest predictive power (21, 45). In addition, another study found that although ABSI was positively associated with arterial stiffness, its AUC value was significantly lower than WHtR in differentiating arterial stiffness, suggesting that ABSI might not be a better predictor of arterial stiffness in Chinese population (19). Besides, A large European cohort study found that WC, BMI and WHtR were J-shaped correlated with all-cause mortality, while ABSI was positively associated with all-cause mortality, and BMI was superior to ABSI in predicting CVDs mortality (46). Furthermore, a meta-analysis of 30 clinical studies showed that higher ABSI was associated with increased risks of hypertension, type 2 diabetes, CVDs and all-cause death, which increased by 13, 35, 21, and 55%, respectively, and ABSI was superior to WC and BMI in predicting all-cause mortality, but it performed poorly in predicting chronic diseases (23). And a previous study has found that ABSI was associated with depression and anxiety, but this correlation existed only in men (47). In addition to these diseases, Zhang et al. have shown that higher ABSI was closely associated with higher urinary albumin-creatinine ratio (48). However, the studies mentioned above are aimed at exploring the association between ABSI and other diseases, and there is little evidence to compare ABSI with other anthropometric indicators in predicting the risk of AAC. Our study was the first to determine the ability of ABSI to recognize AAC. The results showed that higher ABSI increased the risk of AAC by 20–30% compared with lower ABSI. Additionally, we found that ABSI was a better indicator of AAC than BMI, WC and WHtR, and it showed similar predictive power to baseline risk models in the American population. Moreover, we also found for the first time that participants with higher ABSI had a higher risk of developing AAC in the subgroups with HbA1c < 6.5% and non-osteoporosis. The reason for this might be that osteoporosis and HbA1c ≥ 6.5 were the interference factors of ABSI risk prediction model, which was also the focus of our future research. The homogenization and differentiation of the above studies may be explained by the differences in race, sample size and population characteristics.

Innovatively, our findings added to the evidence between ABSI and CVDs from clinical to subclinical diseases. Moreover, we compared the predictive value of ABSI and other nutrition indexes for AAC for the first time. Therefore, this study provided additional information that the evaluation of ABSI might be of clinical significance in primary prevention to identify people at risk of CVDs. In spite of this, our study still had several limitations. Firstly, the present study was a cross-sectional study, which could not identify the causal association between ABSI and AAC. Secondly, in multivariate logistic regression analysis, we only controlled for several meaningful confounding factors, but there might be other confounding factors not included in our study, such as inflammatory indicators, menopause of women and use of medications. Thirdly, ABSI with a very small variance was highly concentrated around the mean value, which made it difficult to define the best critical value of ABSI in clinical practice. Fourthly, since the hip circumference of the participants was not measured during NHANES 2013–2014, we were unable to calculate the WHR, which means that we can’t conduct a comparative analysis of ABSI, hip circumference and WHR. Finally, the data of this study only came from the general population of NHANES 2013–2014, so the findings may not be applicable more populations broadly.

## Conclusion

Taken together, our results showed that WC, BMI, WHtR, and ABSI were significantly associated with AAC and found ABSI was superior to BMI, WC, and WHtR in predicting the risk of AAC. However, whether ABSI is suitable for clinical practice needed to be further studied in different populations.

## Preprint statement

A preprint has previously been published (49).

## Data availability statement

The original contributions presented in this study are included in the article/supplementary material, further inquiries can be directed to the corresponding author.

## Ethics statement

The studies involving human participants were reviewed and approved by National Center for Health Statistics of the Center for Disease Control and Prevention Institutional Review Board. The patients/participants provided their written informed consent to participate in this study.

## Author contributions

ZW conceived and designed the study. WL and ZW collected, analyzed and interpreted the data, and drafted the manuscript. WL was responsible for the management and retrieval of data, contributed to initial data analysis and interpretation, revised the article, and embellished the entire article for grammar when the manuscript was revised. WL, ML, JX, JG, and NL revised the manuscript. NL was the designer of the manuscript and approved to submit the manuscript finally. All authors agreed with the order of the author list, the description of the author contributions, read, and approved the final version of the manuscript.
